# Processing-Driven Changes in Phenolic Composition and Antioxidant Functionality of Aronia Snacks: Insights from In Vitro Gastrointestinal Digestion

**DOI:** 10.3390/foods15101657

**Published:** 2026-05-09

**Authors:** Senem Suna

**Affiliations:** Department of Food Engineering, Faculty of Agriculture, Bursa Uludag University, Bursa 16059, Türkiye; syonak@uludag.edu.tr

**Keywords:** aronia, drying, in vitro gastrointestinal digestion, phenolics

## Abstract

The mechanistic impact of drying technologies on phenolic stability and gastrointestinal bioaccessibility in aronia remains poorly defined, limiting the development of functionally optimized dried berry products. This study aimed to comparatively evaluate the effects of different drying techniques—hot air drying (60, 70, and 80 °C), vacuum drying (60, 70, and 80 °C; 150 mbar), and microwave drying (180 and 360 W)—on total phenolic content (TPC), total antioxidant capacity (TAC) assessed by DPPH, CUPRAC, and FRAP, and total monomeric anthocyanins (TMA) during in vitro gastrointestinal digestion. UHPLC-DAD analysis showed that the phenolic profile was dominated by chlorogenic acid, catechin, caffeic acid, epicatechin, and quercetin. Drying enhanced extractable TPC, while TAC with DPPH and FRAP showed increasing trends and CUPRAC decreased after drying. Color changes indicated increased redness and pigment concentration following dehydration. Simulated digestion induced substantial losses in TPC (53–59%) and TMA (30.5–72.8%), alongside marked reductions in FRAP and CUPRAC, whereas DPPH activity increased significantly, suggesting matrix-driven transformation and release of antioxidant compounds under gastrointestinal conditions. Among the applied methods, vacuum drying (70 °C; 150 mbar) exhibited superior stability in terms of antioxidant and anthocyanin preservation during digestion. Overall, the findings demonstrate that drying-induced structural modifications play a key role in governing phenolic stability and bioaccessibility, providing new insights into the mechanisms underlying the functional behavior of dried berry products.

## 1. Introduction

The species *Aronia melanocarpa* (Michx.) Elliott, a deciduous shrub belonging to the Rosaceae family, is native to North America but has successfully adapted to a wide range of European climatic conditions [1]. Within the genus Aronia, it is the most widely cultivated species and is commonly referred to as black chokeberry, a name derived from the fruit’s characteristic astringent taste associated with its high phenolic content [2]. Although the literature provides extensive information on the geographical distribution and production of aronia, such descriptive data offer limited contribution to understanding the effects of processing on its functional properties; therefore, detailed production statistics are not emphasized in this study.

Aronia is widely recognized as one of the richest antioxidant sources among berry fruits due to its complex biochemical composition, including high levels of phenolic compounds such as anthocyanins, flavonols, and phenolic acids. These compounds are responsible for its antioxidant, anti-inflammatory, and antiproliferative activities and have been associated with protective effects against chronic diseases [3,4,5]. However, despite this well-established compositional and functional profile, previous studies have predominantly focused on identifying bioactive compounds or reporting their health effects, while the impact of processing conditions on their stability and transformation remains insufficiently explored. Due to its intense pigmentation and bioactive composition, aronia is widely processed into dried fruits and various value-added products [6,7,8,9]. Nevertheless, existing studies on processed aronia products are largely descriptive and product-oriented, often lacking a mechanistic evaluation of how processing alters phenolic composition and functionality.

Drying is one of the most widely used preservation techniques for transforming perishable fruits into shelf-stable products. As a heat- and mass-transfer process, it contributes to enzyme inactivation and extended shelf life. However, drying may also result in degradation of heat- and oxygen-sensitive compounds such as anthocyanins, which are known to be highly unstable under thermal and oxidative conditions. Despite numerous studies investigating the drying of fruits, most have evaluated single drying methods or focused solely on physicochemical or antioxidant changes, without systematically comparing different techniques under comparable conditions. Moreover, the behavior of phenolic compounds during gastrointestinal digestion after drying has received limited attention.

Among commonly used techniques, hot air drying (HAD), vacuum drying (VD), and microwave drying (MW) present distinct advantages in terms of process efficiency and product quality. HAD is widely applied due to its simplicity [10]. VD enables drying at lower temperatures and pressures, and MW provides rapid and volumetric heating [11,12]. However, the comparative effectiveness of these techniques in preserving phenolic compounds and maintaining their bioaccessibility remains unclear, particularly for aronia.

Therefore, the present study aims to address these limitations by systematically comparing HAD, VD, and MW drying methods in terms of their effects on physicochemical properties, phenolic composition, antioxidant capacity, and in vitro gastrointestinal bioaccessibility of aronia fruits. In doing so, this study fills a critical gap in the literature by linking drying-induced structural changes with the stability and bioaccessibility of phenolic compounds, thereby moving beyond descriptive analyses toward a process–functionality framework.

## 2. Material and Methods

### 2.1. Material

Aronia (*Aronia melanocarpa*) fruits with an initial total soluble solids content of 15.50 g/100 g were obtained from an orchard in Yalova, Türkiye (40.599605 N, 29.237755 E). The fruits were harvested at full maturity (September 2024), characterized by a dark purple–black color. After washing, the berries were stored at −18 °C until use. Fruit width, length and depth were measured as 1.12 ± 0.20, 1.03 ± 0.06, and 1.14 ± 0.05 cm, respectively, with a vernier caliper.

### 2.2. Drying Procedure of Aronia Fruits

**Figure 1:**
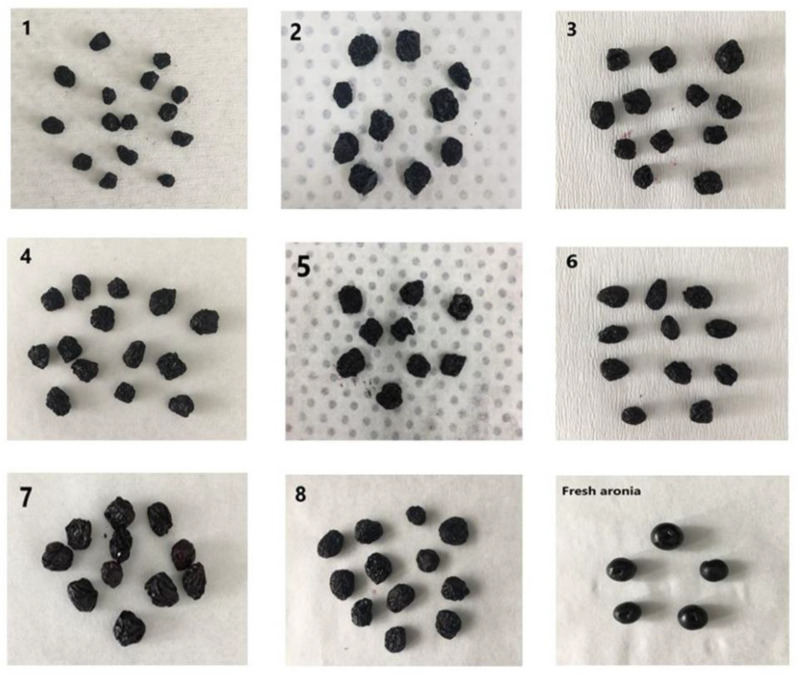
Fresh aronia fruit and the dried samples (1: 80 °C HAD (hot air drying); 2: 70 °C HAD; 3: 60 °C HAD; 4: 80 °C, 150 mbar VD (vacuum drying); 5: 70 °C, 150 mbar VD; 6: 60 °C, 150 mbar VD; 7: 360 W MW (microwave drying); 8: 180 W MW).

Aronia fruits were dried using hot air (HAD), vacuum (VD) and microwave drying (MD); photographs of the fresh aronia fruit and the dried products are given in Figure 1.

In all drying experiments, 5 g of fruit sample was uniformly spread on greaseproof paper and subsequently subjected to the respective drying treatment. Drying experiments were conducted in triplicate. Hot air drying was conducted using a cabinet-type laboratory dryer (Yucebas Machine Analytical Equipment Industry Y35, Izmir, Türkiye) operating at 220 V, 50–60 Hz and 200 W. Temperature and relative humidity inside the dryer were monitored using a temperature sensor (accuracy ±2 °C) and a relative humidity sensor (accuracy ±2%). Drying treatments were carried out at 60, 70, and 80 °C, under a constant relative humidity of 20% and an air velocity of 0.2 m/s. The temperatures were applied in accordance with previous reports [13]. Throughout the drying process, samples were periodically removed, weighed, and then placed back into the dryer. Fruit samples were weighed at 15 min intervals over a duration of 10 h, and moisture loss was calculated using a digital balance (MS3002S, Mettler Toledo, Greifensee, Switzerland.) with an accuracy of 0.01 g. Depending on the drying temperature, the experiments were completed within 360 to 600 min.

Vacuum drying experiments were conducted using a vacuum dryer (VO400, 49 L, Memmert, Schwabach, Germany) at temperatures of 60, 70 and 80 °C under a vacuum pressure of 150 mbar. The temperature and pressure conditions were selected based on [14]. Moisture loss was monitored at 15 min intervals over a total period of 9 h. The drying duration ranged from 200 to 555 min, depending on the applied temperature and vacuum pressure.

Microwave drying experiments were carried out using a domestic microwave oven (Bosch, HMT72G420, Munich, Germany) operating at 230 V–50 Hz with a maximum output power of 800 W. The microwave cavity measured 520 × 479 × 341 cm and included a rotating glass plate with a diameter of 315 mm at its base. Drying processes were performed at microwave power levels of 180 W and 360 W. The drying time varied between 80 and 320 min, depending on the applied power level. Throughout the drying process, the glass plate was removed and weighed at intervals of 1 min at 180 W and 0.5 min at 360 W using a digital balance (Mettler Toledo, MS3002S) with an accuracy of 0.01 g.

A moisture analyzer (MA150, Sartorius, Göttingen, Germany) was employed to determine the moisture content of the dried fruit samples. The drying process was sustained until the moisture level was reduced to approximately 0.07 g water/g dry basis, from an initial value of 2.22 g water/g dry basis. During each drying treatment, sample weight measurements were performed with a maximum duration of 10 s.

### 2.3. Mathematical Modeling of Drying Curve

Twelve thin-layer mathematical drying models were utilized to choose the best model for portraying the drying curve of aronia fruits. Page, Modified Page, Logaritmic, Lewis, Henderson and Pabis, Wang–Singh, Weibullian I, Weibullian II, Midilli I, Modified Midilli I, Modified Midilli II and Aghbashlo models were applied according to the previous literature as described by Öksüz and Buzrul [15]. Root-mean-square error (RMSE) quantified the deviation between predicted and observed values. Model performance in hot air (HAD), vacuum (VD) and microwave (MD) drying was evaluated using a higher coefficient of determination (R^2^) and reduced RMSE and chi-square (χ^2^) values as indicators of better fit, with all statistical expressions taken from Suna [11].

### 2.4. Analysis Methods

#### 2.4.1. Physicochemical Analyses and Color Attributes

Total soluble solids (°Brix) were determined with a digital refractometer (RA-500N, KEM, Tokyo, Japan), based on refractive index measurements, and moisture content was quantified by oven-drying in accordance with AOAC [16] guidelines. Color attributes of aronia fruits and dried samples, including L*, a*, b*, chroma (C*), and hue angle (h°), were measured using a chromameter (CR-5, Konica Minolta, Osaka, Japan) [17].

#### 2.4.2. Extraction of Bioactive Compounds from Undigested Samples

Extraction of bioactive constituents was carried out following Deniz and Suna [18]. The resulting supernatants were stored at −18 °C until analysis of total phenolic content (TPC), total antioxidant capacity (TAC), and total monomeric anthocyanins (TMA).

#### 2.4.3. In Vitro Gastrointestinal Digestion Procedure

Aronia fruit and dried samples were subjected to in vitro gastrointestinal digestion following the INFOGEST protocol [19,20]. Simulated salivary fluid (SIF), gastric fluid (SGF) and intestinal fluid (SIF) were prepared as specified. Initially, 5 g of each sample was mixed with 3.5 mL of SIF (pH = 7) and 25 μL of 0.3 M CaCl_2_, and the total volume was topped up with distilled water to 10 mL. For the gastric phase, samples were mixed with 7.5 mL of SGF, and the pH was adjusted to 3 using 1 M HCl. Then 1.6 mL of porcine pepsin (25,000 U/mL) and 5 μL of 0.3 M CaCl_2_ were added, and the final volume was adjusted to 20 mL with distilled water. The mixtures were then incubated for 2 h at 37 °C.

The intestinal phase involved adding SIF (11 mL), pancreatin (800 U/mL) (5 mL), bile extract (160 mM) (2.5 mL), and 40 μL of 0.3 M CaCl_2_ to the mixture. The pH was then adjusted to 7.0 with NaOH. The final volume was made up to 40 mL with distilled water, and then the mixtures were incubated in a water bath at 37 °C for 2 h. Finally, digesta were centrifuged at 3500 rpm for 10 min, and supernatants were filtered, combined, cooled to 4 °C to stop enzymatic activity, and stored at −18 °C until analysis.

#### 2.4.4. TPC Analysis

Spectrophotometric analyses were conducted using a Shimadzu UV-1208 (Japan). The TPC of undigested and digested samples was determined following Kocer et al. [21] and expressed as mg of gallic acid equivalent (GAE)/100 g dry weight (dw), with an R^2^ of 0.9992.

#### 2.4.5. TAC Analysis

TAC was assessed using three complementary assays: DPPH (2,2-diphenyl-1-picrylhydrazyl radical-scavenging activity; R^2^ = 0.9997) [22], CUPRAC (cupric ion reducing antioxidant capacity; R^2^ = 0.9978) [23], and FRAP (ferric reducing antioxidant power; R^2^ = 0.9934) [24]. Trolox^®^ (6-hydroxy-2,5,7,8-tetramethylchroman-2-carboxylic acid) was used as the standard. All analyses were performed in triplicate, and results were expressed as µmol Trolox equivalent (TE)/g dw.

#### 2.4.6. TMA Analysis

TMA in aronia fruit and pestil samples was quantified using the AOAC [25] pH-differential method and expressed as cyanidin-3-glucoside equivalents (mg/kg).

#### 2.4.7. Determination of Phenolic Compounds of Aronia Fruit by UHPLC-DAD (Ultra-High-Performance Liquid Chromatography–Diode Array Detector)

The method was adapted from Kim et al. [26]. For phenolic extraction, 2 g of aronia sample was placed in falcon tubes, mixed with 10 mL of 70:30 methanol:water, vortexed for 1 min, and sonicated in the dark for 30 min (621.06.0109, Isolab, Eschau, Germany). Samples were centrifuged at 4100 rpm and 20 °C for 25 min (NF 800, Nüve, Ankara, Türkiye), and the extraction was repeated three times. Combined supernatants were filtered through a 0.45 μm PVDF filter (Millex-HV, Merck Millipore, Darmstadt, Germany) and transferred to vials. Phenolic analysis was performed by UHPLC (Dionex Ultimate 3000, Thermo Fisher Scientific, Waltham, MA, USA) with a DAD detector and an Inertsil ODS-3 column (150 × 4.6 mm, 5 µm). The conditions were as follows: 1 mL/min flow rate, 30 °C column temperature, 5 µL injection volume, and a gradient mobile phase of A (0.2% phosphoric acid) and B (acetonitrile:methanol, 50:50). The gradient was 4% B (0 min), 50% B (0–40 min), 60% B (40–45 min), 100% B (45–60 min), 50% B (60–70 min), and 50% B (70–71 min). Single standards (100 mg) were dissolved in methanol, and calibration curves (5–100 ppm) were prepared for catechin (R^2^ = 0.9998), chlorogenic acid (R^2^ = 0.9997), caffeic acid (R^2^ = 0.9999), epicatechin (R^2^ = 0.9998), and quercetin (R^2^ = 0.9999) (Sigma-Aldrich, St. Louis, MO, USA). Quantification was performed at 325 nm for chlorogenic acid and 280 nm for the remaining standards.

#### 2.4.8. Statistical Analysis

All measurements were conducted in triplicate, and data are presented as mean ± standard deviation.

One-way analysis of variance (ANOVA) was performed to evaluate significant differences among groups at *p* < 0.05. Following a significant ANOVA result, Tukey’s honestly significant difference (HSD) post hoc test was applied to determine differences between group means. All statistical analyses were conducted using JMP software (Version 8.0, SAS Institute Inc., Cary, NC, USA). Principal component analysis (PCA) was carried out with Minitab statistical software (Minitab Inc., State College, PA, USA, Version-10).

## 3. Results and Discussion

### 3.1. Physicochemical Properties and Color Changes

The weight, total dry matter (g/100 g) and total soluble solids (TSS) of aronia fruits were 1.05 ± 0.07 g, 24.00 ± 0.30 g/100 g and 17.50 ± 0.19 g/100 g, respectively. The weight of our fruits was found to be in good agreement with a previous study which reported a weight of 1.04 g [6]. The TSS value (17.50 ± 0.19 g/100 g) was consistent with previously reported ranges (16–21 g/100 g), while the dry matter content (24.00 ± 0.30 g/100 g) similarly fell within the literature range of 24.4–29.2 g/100 g [27]. Color values of the fresh fruit and the dried products are given in Table 1.

The L* value of fresh aronia (21.36 ± 0.66) was found to be similar to previously reported values of 24.06 [7] and 24.64 [6]. Color variability is typically influenced by cultivar, harvest maturity, and origin. All dried fruits showed significantly lower brightness (L*) than the fresh fruit (*p* < 0.05), with decreases of 19.93 (HAD 80 °C)–46.34 (HAD 60 °C) %, indicating a general darkening of the samples irrespective of the technique applied. Dried aronia fruit L* values (11.46–17.10) (Table 1) were also consistent with the figure of 18.56 reported by Górska-Horczyczak et al. [6] in convection drying at 65° for 24 h, who reported a decrease between fresh and dried aronia fruits.

Within the HAD process, an inverse relationship was observed between temperature and lightness: L* increased as the drying temperature rose from 60 °C to 80 °C (11.46 to 17.10). Although higher temperatures are often associated with thermal browning, the darker appearance at lower temperatures in this study may be attributed to prolonged drying times, which extend the exposure of anthocyanins to enzymatic and oxidative degradation. Thus, despite lower thermal intensity, the cumulative thermal and oxidative load at extended drying durations likely enhanced pigment breakdown, yielding lower L* values. A comparison between HAD and vacuum drying (VD) at equivalent temperatures revealed that vacuum treatment did not consistently preserve lightness. At 60 °C, VD resulted in a higher L* value (12.18) than HAD (11.46), consistent with the expected protective effect of reduced oxygen availability on color degradation. However, at 70 °C and 80 °C, VD samples exhibited lower L* values than their HAD counterparts. This deviation may stem from structural collapse caused by rapid moisture removal under reduced pressure, in combination with the heightened thermal sensitivity of anthocyanins at elevated temperatures. Additionally, drying under vacuum often requires longer processing times, which could further intensify overall color deterioration despite lower oxygen levels.

Microwave drying (MW) produced comparatively higher L* values (16.00–16.54), reflecting better color retention relative to most HAD and VD treatments. This outcome is consistent with the nature of microwave heating, where volumetric energy absorption accelerates water removal and minimizes the duration of oxidative and enzymatic reactions responsible for darkening. As a result, anthocyanin degradation is reduced, and the samples retain a lighter appearance. Overall, the findings suggested that lightness loss is inevitable during the dehydration of anthocyanin-rich fruits; increasing temperature in HAD reduces total exposure time and thereby enhances color preservation, vacuum drying provides effective color protection only at lower temperatures, and microwave drying offers the greatest preservation of L* due to rapid moisture removal and minimal oxidative exposure.

The a* value of fresh aronia (0.06 ± 0.01) was much lower than the 2.56 reported by Górska-Horczyczak et al. [6] and the 14.29 reported by Olcay et al. [7]. All drying treatments led to a substantial increase in a* compared to fresh aronia fruit, indicating a general shift toward more reddish hues upon dehydration (*p* < 0.05). Dried aronia fruit a* values (0.18–0.88) (Table 1) were also consistent with the 2.36 reported by [6] in convection drying at 65° for 24 h, who reported a decrease of 7.03% between fresh and dried aronia fruits. This observation aligns with prior research showing that drying concentrates anthocyanin pigments and can enhance chromatic intensity through pigment concentration and limited polymerization [28]. Within the HAD process, the most pronounced increase in a* was observed at 60 °C, with values decreasing at higher temperatures (70–80 °C). When interpreted alongside the corresponding L* trends—where 60 °C HAD produced the darkest samples—these results suggest that prolonged drying under lower temperatures may promote extended oxidative and enzymatic reactions, driving pigment transformation into polymeric or brownish derivatives while increasing redness [29]. In contrast, higher temperatures shorten the drying time, reducing the duration of oxidative stress and resulting in comparatively lower a* values despite higher thermal exposure [30]. Comparison of HAD and vacuum drying (VD) at the same temperature revealed consistently higher a* values under VD, suggesting that reduced oxygen conditions can partially preserve or intensify anthocyanin-associated redness by limiting complete oxidative bleaching, an effect similarly reported in berry drying studies [12]. However, VD did not always preserve lightness (L*), indicating that vacuum benefits are counteracted by thermal sensitivity and structural changes at elevated temperatures. Microwave drying also produced elevated a* values, which, combined with shorter exposure times and rapid moisture loss, contributed to better pigment retention compared to longer convection-based drying [28].

The b* value of fresh aronia (−0.02 ± 0.01) decreased markedly in dried fruits −1.02 (80 °C, 150 mbar VD) to −0.33 (360 W MW), indicating a near-neutral color balance between blue and yellow due to thermal degradation of heat-sensitive pigments (*p* < 0.05) [31]. Negative b* values, together with positive a* values, confirm the shift toward a purplish–blue hue. This trend aligns with observations reported in other studies on anthocyanin-rich fruits, in which b* values often decrease due to degradation and transformation of yellow–blue chromophores as a result of pigment oxidation and polymerization during dehydration. For example, studies on goji and other berries have shown significant decreases in b* values after hot air drying, reflecting a reduction in yellowness and increased blue–purple tonality [29].

When considering the effect of temperature within HAD, the magnitude of the decrease in b* values was more pronounced at higher temperatures. At 80 °C, b* values shifted toward more negative levels compared to lower drying temperatures, indicating a stronger reduction in yellowness and a greater tendency toward blue–purple tones. In contrast, samples dried at 60 °C exhibited a more moderate decline relative to the fresh fruit, suggesting less pronounced color alteration. This trend may be linked to the combined effects of thermal degradation of anthocyanins and shorter exposure times at higher temperatures. Higher temperatures accelerate drying, reducing the time for enzymatic browning but increasing thermal stress, which can destabilize pigment structures and shift hue values further toward the blue–purple region [29,32]. Conversely, at lower temperatures, the slower dehydration process prolongs the exposure period, which may preserve certain pigment-associated yellow components slightly better, resulting in less negative b*.

Furthermore, when comparing HAD to VD at equivalent temperatures, a clear pattern emerged. At the same drying temperature, VD samples generally exhibited more negative b* values than HAD samples. At comparable drying temperatures, vacuum-dried samples often show better retention of color parameters including b* compared to hot-air-dried samples, likely due to the reduced oxygen environment slowing oxidative degradation of pigments. For example, vacuum drying better preserved b* values in celeriac slices relative to hot air drying at 65 °C, consistent with reduced pigment loss under low-oxygen conditions. This phenomenon can be attributed to the reduced oxygen environment during VD, which not only slows oxidative degradation of anthocyanins but also encourages the formation of polymeric pigments with blue–purple hues [32]. However, vacuum conditions can also amplify structural collapse of fruit tissue, potentially exposing pigments more directly to residual heat and causing further shifts in color parameters [33].

Chroma (C*), reflecting color intensity, was 0.06 ± 0.02 for fresh fruit, similar to previous observations that C* varies with maturity [34]. Dried fruits’ C* ranged from 0.72± 0.04 to 1.15 ± 0.03 (*p* < 0.05) (Table 1). Hue angle (h°) values of dried fruits were significantly lower than those of the fresh fruit (*p* < 0.05), with ratios of 0.72 (360 W MW) to 14.81 (70 °C HAD) % (Table 1). In comparison to the fresh samples, the chroma (C*) and hue (h°) values of dried aronia exhibited considerable alterations across all drying conditions due to moisture removal and pigment transformation. Generally, chroma decreased relative to the fresh fruit, reflecting not only pigment concentration but also notable anthocyanin degradation and oxidation, which result in less vivid coloration during drying. Similar trends have been observed in anthocyanin-rich fruits, where dehydration induces color loss due to thermal or oxidative stress. Regarding the influence of temperature, higher drying temperatures accelerated pigment decomposition, causing greater reductions in chroma and shifts in hue values. Elevated temperatures destabilize anthocyanins and promote the formation of brownish or polymerized compounds, thus diminishing color purity and altering hue direction, as established in studies examining anthocyanin thermal degradation [35].

When a vacuum was applied at the same temperatures, chroma values were generally better preserved compared to hot air drying, likely due to lower oxygen exposure mitigating oxidative breakdown. However, vacuum drying sometimes induced structural collapse and extended process durations, which also contributed to hue shifts associated with browning. The literature indicates that vacuum conditions can partially retard oxidation but may not completely prevent thermal degradation, especially at higher temperatures [36].

### 3.2. Phenolic Profile of Aronia Fruit

**Figure 2:**
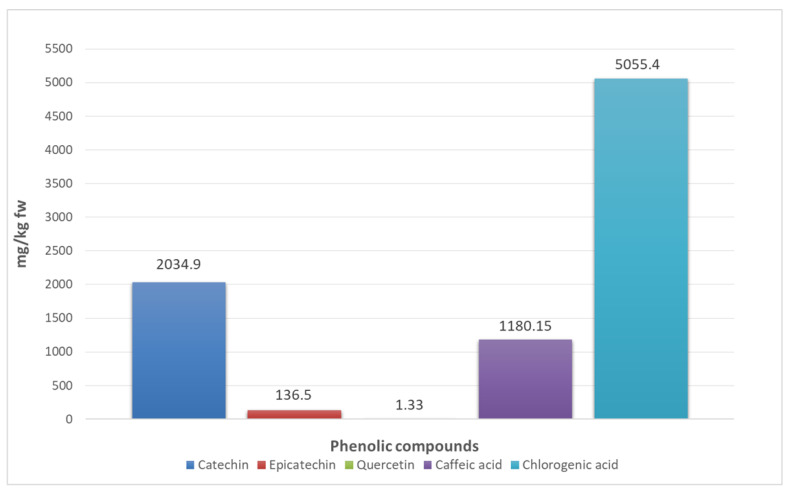
Individual phenolics of aronia fruit. Results are given as mean ± standard deviation (n = 3). fw: fresh weight.

Figure 2 presents the phenolic profile of aronia fruit, characterized by high levels of chlorogenic acid (5055.40 ± 171.76 mg/kg), catechin (2034.90 ± 107.82 mg/kg), caffeic acid (1180.15 ± 38.93 mg/kg), epicatechin (136.50 ± 3.39 mg/kg), and quercetin (1.33 ± 0.05 mg/kg). As with many berries, aronia contains abundant phenolic acids—mainly hydroxylated benzoic and cinnamic acid derivatives—and flavonoids such as flavonols and flavanols. In the current classification of phenolic compounds, catechin and epicatechin are categorized as flavanols within the flavonoid group, while quercetin is classified as a flavonol. Additionally, caffeic acid and chlorogenic acid are grouped under hydroxycinnamic acids within the phenolic acids subclass.

Aronia notably includes hydroxycinnamic derivatives like caffeic and caffeoylquinic acids. Caffeic acid plays a key role in neutralizing reactive oxygen species, while caffeoylquinic acids—formed via the shikimate–phenylpropanoid pathway—exhibit strong antioxidant, anti-inflammatory, and hepatoprotective activities. Chlorogenic acid (3-O-caffeoylquinic acid), the major isomer, is widely regarded as a biochemical marker of antioxidant capacity [37].

Flavonoid constituents include flavanols (catechin and epicatechin) and flavonols (quercetin derivatives). Catechins have been associated with neuroprotective and bioactive effects, including potential roles in modulating metabolic and cognitive functions, particularly in neurodegenerative disorders [38]. Quercetin—abundant in berries [39]—is widely studied for its anti-obesity, antimicrobial, anticancer, and anti-inflammatory properties [40]. Consistent with the present findings, earlier studies also identified chlorogenic acid as the predominant phenolic in *Aronia melanocarpa*, with lower quercetin levels [41]. Denev et al. [42] reported chlorogenic acid (963–1879 mg/kg fw) and epicatechin (734–1240 mg/kg fw) as major constituents, while quercetin ranged widely (65–298 mg/kg fw). Gerasimov et al. [43] likewise observed chlorogenic acid (13–99 mg/kg fw), epicatechin (17–83 mg/kg fw), and quercetin (1–4 mg/kg fw), with our results generally higher—especially for chlorogenic acid.

Additional comparisons showed good agreement: Tian et al. [44] reported 1644.20 mg/kg fw chlorogenic acid; Yang et al. [45] measured 189 mg/kg dw caffeic acid; Oszmiański and Lachowicz [46] reported 1227 mg/kg dw catechin; and Oszmiański and Wojdyło [47] documented 3018.5 mg/kg dw chlorogenic acid and 150.4 mg/kg dw epicatechin. Rop et al. [48] reported chlorogenic acid (1131–1960 mg/kg) and epicatechin (467–862 mg/kg), also generally lower than our findings.

Overall, the literature confirms substantial variability in the phenolic composition of A. melanocarpa across cultivars, growing conditions, and analytical methods. Nonetheless, quercetin tends to remain relatively stable, whereas chlorogenic acid and epicatechin show greater sensitivity to environmental and processing factors.

### 3.3. Total Phenolics of Aronia Fruit, Dried Fruits and Their In Vitro Bioaccessibility

Aronia is widely regarded as a super fruit due to its high phenolic content. In this study, the TPC of fresh aronia was 640.41 ± 4.14 mg GAE/100 g dw (Table 2).

Reported values in the literature (856.38–1205.57 mg GAE/100 g fw (Croatia) [49], 930.4 mg GAE/100 g fw [50], 7967 mg GAE/100 g dw (Poland) [8] and 13,550 mg GAE/100 g dw (Korea) [26]) vary considerably due to differences in cultivar, climate, ripeness, extraction and analytical procedures, processing methods, and storage conditions.

TPC declined slightly (1.01–5.25%) following different drying processes. These relatively small changes indicate that phenolic stability is governed by a balance between enzymatic oxidation and thermal degradation mechanisms. At higher temperatures (70–80 °C), rapid inactivation of polyphenol oxidase and peroxidase limits enzymatic oxidation, thereby preserving phenolic compounds. In contrast, at lower temperatures or prolonged drying times, continued oxygen exposure may promote oxidative degradation [51]. Even 60 °C HAD led to only about a 1% reduction, confirming that hot air drying effectively preserves phenolic compounds. In another study, a 2.51% decrease between fresh and dried aronia berries was reported [6]. Higher drying temperatures yielded higher TPC, consistent with findings in dried aronia [52]. This effect is typically attributed to shorter drying times at higher temperatures, reducing oxidative degradation. Similar reductions have been reported in various thermally processed berry products [53]. Changes in fruit matrix composition, enzyme activity, and extraction conditions further influence extractable TPC.

Under vacuum drying (150 mbar), reductions remained limited: 1.5–2% at 70 °C and 60 °C, and around 3% at 80 °C. This can be mechanistically explained by reduced oxygen availability, which suppresses oxidation reactions. However, the lower boiling point under vacuum may enhance cell structure disruption, influencing extractability rather than true retention of phenolics. The most noticeable decreases occurred in the microwave-dried samples, where 360 W (5.25%) and 180 W (4.98%) resulted in greater losses compared to other methods. This behavior is attributed to rapid and non-uniform heating, which can induce localized thermal degradation, structural breakdown, and polymerization reactions of phenolic compounds.

Overall, phenolic retention is not solely dependent on temperature but is strongly influenced by the interaction between heat transfer kinetics, oxygen availability, and food matrix disruption [51].

In this study, the TPC of dried aronia fruits ranged between 606.85 ± 6.70 and 644.73 ± 2.48 mg GAE/100 g dw (Table 2) (*p* < 0.05). We found lower values of TPC in dried *Aronia melanocarpa* fruits compared to 1954–2466 mg GAE/100 g [51], 5315 mg GAE/100 g dw (in HAD-dried chokeberry at 50 °C) [54], 6354 mg GAE/100 g dw (in VD-dried chokeberry at 50 °C 60 mbar) [55], 7767 mg GAE/100 g dw (by convective drying at 65 °C for 24 h) [6], and 79,230 mg GAE/100 g dw (by oven drying at 60 °C for 12 h) [56], being strongly dependent on the drying method and thermal load.

This relatively high retention suggests that moderate convective heating is sufficient to inactivate polyphenol-oxidizing enzymes while avoiding excessive thermal degradation. More variable outcomes highlight the combined effects of temperature, drying kinetics, and oxygen exposure on phenolic stability. Together, these findings indicate that while hot air drying at moderate temperatures (60–70 °C) is generally effective in maintaining phenolic integrity, vacuum drying or lower-temperature approaches may yield more heterogeneous outcomes. This comparative evidence reinforces that optimal retention of bioactive compounds in dried aronia requires careful control of both heat intensity and process atmosphere. Additionally, the TPC of several aronia-based products like aronia fruit leather was reported as 2043 mg GAE/100 g [7], while aronia fruit tea, powders and juices were determined to be within the range of 1494–3436, 4233–4951 and 3002–6639 mg GAE/100 g dw, respectively.

Gastric digestion reduced TPC, with 180 W MW and 80 °C, 150 mbar VD showing the lowest and highest changes, respectively. The sharp decrease during gastric digestion (68.19–70.80%) is primarily associated with the acidic environment (pH ~2), which promotes hydrolysis, structural destabilization, and interactions of phenolics with proteins and other macromolecules, thereby reducing their extractability [11,55,57]. During the intestinal phase, although partial recovery relative to the gastric phase was observed, TPC remained substantially lower (53–59%) than in undigested samples. This behavior indicates that alkaline conditions further induce degradation, oxidation, and transformation of phenolics into smaller or structurally modified compounds that may not be fully detected by the Folin–Ciocalteu assay [58]. The greatest decrease occurred in the 60 °C HAD sample (58.76%), while the smallest decrease was observed in the 360 W microwave treatment (53.76%). Tomas et al. [50] reported a substantial reduction in aronia fruit, with TPC values decreasing from 930.4 mg GAE/100 g prior to digestion to 452.6 mg GAE/100 g following digestion. All processing methods also showed a substantial decline in phenolic content during intestinal digestion, consistent with findings for cherry laurel extracts [59] and pear ferment [57]. This reduction is chiefly attributed to the compromised stability of phenolics when subjected to enzymatic activity and dynamic shifts in pH during digestion [60]. Overall, these results demonstrate substantial phenolic loss during drying and digestion and highlight the strong effect of processing conditions on the stability and gastrointestinal release of dietary polyphenols [61].

### 3.4. Total Antioxidant Capacity of Aronia Fruit, Dried Fruits and Their In Vitro Bioaccessibility

In this study, the TAC of aronia, dried fruits, and their in vitro bioaccessibility was evaluated by DPPH, CUPRAC, and FRAP assays (Table 2). TAC assays are based on different reaction mechanisms. DPPH reflects hydrogen atom transfer (radical scavenging), whereas CUPRAC and FRAP are based on electron transfer reactions. Therefore, differences among these assays are expected, and they should be interpreted as complementary rather than directly comparable. Accordingly, polyphenols, particularly anthocyanins, contribute to antioxidant activity through radical scavenging and redox reactions [5].

The TAC of fresh aronia by DPPH was 168.97 ± 2.09 µmol TE/g dw (Table 2). The literature values span a wide range depending on cultivar, origin, and basis, for example 106.5 µmol TE/g dw [62], and 289.5 µmol TE/100 g dw [63]. Although direct comparison is limited by unit differences, our results fall within the reported interval. Processing into dried fruits reduced DPPH by 91.57–93.19%. This significant reduction indicates that radical-scavenging compounds, particularly anthocyanins, are highly sensitive to thermal degradation, structural transformation, and polymerization during drying. Miletić et al. [64] likewise reported lower DPPH in dried aronia (18.15 µmol TE/g dw), and Ceylan et al. [54] reported a decrease of 13.09% in VD and 25.18% in HAD-dried aronia fruits compared to fresh fruits. In the present study, the DPPH radical-scavenging capacity of dried aronia fruits ranged from 11.51 ± 0.40 to 14.24 ± 0.13 µmol TE/g dw (Table 2) (*p* < 0.05). These values fall within the broad variability previously documented in the literature. For instance, Ceylan et al. [54] reported DPPH activities of 67.11 mg TE/g dw for HAD-dried chokeberries at 50 °C and 77.96 mg TE/g dw for VD-dried samples processed at 50 °C and 60 mbar. Similarly, Górska-Horczyczak et al. [6] observed a DPPH inhibition of 58.91% in aronia fruits convectively dried at 65 °C for 24 h, while Thi and Hwang [56] reported 75–80% inhibition for fruits subjected to oven drying at 60 °C for 12 h. Tolić et al. [49] further documented DPPH capacities ranging from 183.52 to 191.31 mmol TE/100 g dw in dried aronia berries, and Kapci et al. [65] reported DPPH of dried aronia fruits as 30.5 and 36.3 g TE/kg. Collectively, these findings highlight the substantial variability in antioxidant activity depending on the drying method and processing conditions.

For fresh aronia, the CUPRAC value was 825.24 ± 92.22 µmol TE/g dw, comparable to the literature data [62]. The CUPRAC of aronia was previously reported by Ceylan et al. [54] and Kapci et al. [65] as 96.12 mg TE/g dw and 67.7 g TE/kg, respectively. For fresh aronia, the FRAP value was 419.32 ± 12.09 µmol TE/g dw (Table 2), consistent with the figure of 152.3 mM TE/100 g dw previously reported by Taneva [63]. Following the drying treatments, FRAP values exhibited a substantial decline, ranging from 46.13% to 78.79%. However, CUPRAC measurements demonstrated a notable enhancement, with increases of 51.14% to 79.16% when comparing dried samples to the fresh fruit. (Table 2). Similar increases in CUPRAC value from fresh fruit (67.7 g TE/kg) to dried aronia samples (233.2 and 257.2 g TE/kg) were also observed from Kapci et al. [65]. Such reductions are mainly attributed to thermal degradation and oxidation of bioactive compounds under high-temperature conditions [11,66]. The divergence between CUPRAC and FRAP responses can be explained by differences in their reaction principles. The increase in CUPRAC values may result from the formation of Maillard reaction products and the release of bound phenolics with strong electron-donating capacity, whereas the decrease in FRAP values reflects the degradation of ferric-reducing antioxidants, particularly low-molecular-weight phenolics. These findings indicate that antioxidant responses are assay-dependent and reflect different subsets of compounds rather than a uniform antioxidant behavior.

In dried aronia fruits, the CUPRAC ranged from 1246.91 ± 159.86 µmol TE/g dw to 1366.32 ± 176.67µmol TE/g dw, while the FRAP ranged from 88.93 ± 8.34 to 225.87 ± 22.74 µmol TE/g dw (*p* < 0.05). The literature also reported 22.05 mg TE/g dw (in HAD-dried chokeberry at 50 °C) and 51.67 mg TE/g dw (in VD-dried chokeberry at 50 °C, 60 mbar) [54] for CUPRAC, and 17.4–21.4 mmol/100 g of dw for FRAP assays [49]. These values were also broadly comparable with reported TAC ranges for several fruit-based matrices assessed by CUPRAC and FRAP assays [10,59].

In terms of the vacuum application effect on the TAC of aronia fruits, vacuum drying at 150 mbar (VD), compared to hot air drying (HAD) at equivalent temperatures (80, 70, and 60 °C), was investigated through DPPH, CUPRAC and FRAP assays (Table 2). In the DPPH assay, VD consistently yielded lower DPPH compared to HAD at the same temperature. At 80 °C, DPPH activity decreased from 13.19 ± 0.35 µmol TE/g (HAD) to 11.97 ± 0.09 µmol TE/g (VD), indicating a reduction of approximately 9.3%. Similar trends were observed at 70 °C (11.76 ± 0.76 vs. 11.51 ± 0.40 µmol TE/g) and 60 °C (13.41 ± 0.18 vs. 12.03 ± 0.09 µmol TE/g). These results suggested that vacuum conditions may lead to a loss or alteration of certain radical-scavenging compounds, possibly due to volatile phenolic degradation or conformational changes that reduce electron-donating ability. CUPRAC results also exhibited a comparable pattern in which VD also tended to diminish reducing capacity relative to HAD. At 80 °C, CUPRAC values were slightly lower under a vacuum (1330.63 ± 68.29 µmol TE/g) compared to HAD (1366.32 ± 176.67 µmol TE/g). At 70 °C and 60 °C, the reductions were more pronounced, with CUPRAC values decreasing from 1365.27 ± 176.96 to 1252.85 ± 108.90 µmol TE/g and from 1451.84 ± 354.18 to 1309.23 ± 203.80 µmol TE/g, respectively. These findings indicated that the combination of reduced pressure and thermal exposure may limit the formation or stability of reductive antioxidants that contribute to cupric ion reduction.

In contrast to the relatively uniform declines observed in DPPH and CUPRAC assays, the FRAP assay demonstrated a temperature-dependent response to vacuum application. At 80 °C and 70 °C, FRAP values under vacuum (147.68 ± 14.21 and 143.93 ± 14.73 µmol TE/g, respectively) were markedly lower than their HAD counterparts (166.41 ± 17.22 and 225.87 ± 22.74 µmol TE/g). These reductions suggested that VD at elevated temperatures may accelerate the breakdown of ferric-reducing components or inhibit their formation. Notably, at 60 °C, vacuum drying produced a higher FRAP value (148.56 ± 24.35 µmol TE/g) than HAD (116.78 ± 19.20 µmol TE/g), indicating that at lower temperatures, a vacuum may preserve or even enhance certain ferric-reducing antioxidants by limiting oxidative degradation. Overall, differences in TAC results highlight the complex interactions between drying temperature, pressure, and the stability of specific antioxidant compounds while emphasizing that optimization of drying parameters must carefully consider both thermal and pressure effects to maximize the retention of bioactive antioxidant constituents in dried berry products.

When the TAC change at different temperatures (60 °C to 70 °C and further to 80 °C) is considered, it was reported that the TAC of aronia fruit exhibited method-dependent and non-linear responses. For the DPPH assays, an initial decrease in activity was observed between 60 °C and 70 °C, with a partial recovery at 80 °C, indicating that moderate thermal exposure may transiently diminish radical-scavenging compounds but that a higher temperature partially compensates through matrix changes or release of bound antioxidants (Table 2) [52]. A similar pattern emerged in the CUPRAC assay, where values declined from 60 °C to 70 °C but stabilized or slightly increased at 80 °C under both HAD and VD conditions, reflecting the complex balance between thermal degradation and time-dependent oxidative reactions [13]. In contrast, the FRAP assay demonstrated a pronounced increase at 70 °C under HAD, followed by a decrease at 80 °C, whereas VD maintained relatively stable FRAP values across the temperature range, with a modest peak at the lower temperature. Overall, increasing the drying temperature did not result in a simple monotonic increase or decrease in TAC; instead, temperature effects varied by assay and drying condition, reflecting the complex thermal sensitivity of different antioxidant components in aronia fruit. Moderate temperature elevation led to a reduction in TAC as measured by DPPH and CUPRAC, while FRAP reached its maximum at intermediate temperatures, underscoring the importance of optimizing thermal parameters to preserve bioactive compounds.

For the microwave drying application (MW) on aronia samples, the TAC varied according both to the microwave power and the applied assay (Table 2). For DPPH, increasing the microwave intensity from 180 W to 360 W slightly improved radical quenching ability. Shorter drying times, formation of Maillard reaction products, caramelization compounds, and oligomeric phenolic derivatives may enhance radical-scavenging capacity [67]. Conversely, in the CUPRAC, the lower microwave power (180 W) exhibited the highest value among all samples, whereas 360 W resulted in a reduction in reducing power, indicating that moderate microwave power favors the preservation of reductive antioxidants. In the FRAP assay, increasing the MW power decreased TAC, with the 360 W sample showing the lowest FRAP value overall. These patterns suggested that while MW drying can preserve certain antioxidant activities, higher power levels may accelerate degradation of specific phenolic compounds responsible for reducing capacity.

The impact of simulated gastrointestinal digestion on TAC is summarized in Table 2 (*p* < 0.05). Gastrointestinal digestion induced significant, assay-dependent changes in TAC. DPPH showed severe gastric losses (≈49–97%) followed by marked intestinal increases in dried samples (≈178–384%), whereas CUPRAC and FRAP activities experienced substantial reductions during both the gastric (≈68–94% and ≈17–86%, respectively) and intestinal phases, with limited or no recovery. The increase in DPPH during the intestinal phase can be attributed to the release and transformation of phenolic compounds during digestion. While acidic gastric conditions (pH ~2) reduce activity due to protonation and instability, the intestinal phase promotes matrix breakdown and enzymatic hydrolysis, releasing bound phenolics. These may further convert into smaller, more reactive metabolites with higher hydrogen-donating capacity, while deprotonation at near-neutral pH enhances their reactivity [19,20]. These findings demonstrate that antioxidant bioaccessibility after digestion strongly depends on both the analytical method and processing conditions. The present findings are in good agreement with previous studies. In particular, the DPPH trend observed in this study, characterized by a pronounced decrease during the gastric phase followed by a further reduction in the intestinal phase compared with undigested samples, is consistent with the results reported by Tomas et al. [68]. Similarly, the decline in FRAP values after gastric digestion is consistent with previous studies reporting a substantial reduction in antioxidant capacity during gastrointestinal digestion [69]. Moreover, Tomas et al. [50] reported a marked decrease in CUPRAC values, from 1307.4 mg TE/100 g fw before digestion to 759.9 mg TE/100 g fw after digestion, further supporting the digestion-induced loss of reducing capacity observed in the present study.

Compared to the undigested state, CUPRAC and FRAP values declined during the intestinal phase, in agreement with previous reports on medlar, cherry laurel and loquat fruit matrices [11,17,55,70]. This decrease has been attributed to simultaneous reductions in TPC and TMA [71], digestion-induced structural changes that modify antioxidant reactivity and may result in an underestimation of total antioxidant capacity as well as the instability and degradation of antioxidants under alkaline intestinal conditions [72].

### 3.5. Total Monomeric Anthocyanin of Aronia Fruit, Dried Fruits and Their In Vitro Bioaccessibility

Aronia fruits show considerable variation in total and individual polyphenols due to methodological, environmental, and genetic factors, including cultivar, harvest time, climate, and maturity. Compared with other berries, aronia has a relatively simple anthocyanin profile dominated by cyanidin-based glycosides—mainly cyanidin-3-galactoside and cyanidin-3-arabinoside—which account for over 90% of total anthocyanins [73]. In this study, the TMA of fresh aronia fruit was 2554.12 ± 30.53 mg C3G/kg dw (Table 3). Previous reports range widely, e.g., 1079 mg CGE/kg fw in Turkish aronia fruit samples [50], 2782–6860 mg C3G/kg fw in Bulgarian samples [42], and 4605 mg C3G/kg fw in Italian “Nero” [74], consistent with our findings. The TMA of dried samples ranged between 463.13 ± 15.50 and 1313.89 ± 3.29 mg C3G/kg dw (Table 3).

Tolić et al. [49] reported TMA values in dried *Aronia melanocarpa* berries between 1419 and 1471.7 mg C3G/kg dw, while Thi and Hwang [56] reported the TMA of oven-dried aronia berries (heated at 60 °C for 12 h in a single layer on a food dehydrator tray) as 2231 ± 11.4 mg of C3G/g. Drying also reduced TMA by 48.55–69.79% (Table 3). This significant loss is mainly driven by the inherent instability of anthocyanins, which undergo thermal degradation, oxidation, and structural transformations such as glycosidic bond cleavage and chalcone formation. Similar declines were documented in aronia pestils [7] and vacuum-dried sour cherries [75]. The inherent heat sensitivity of anthocyanins makes them particularly susceptible to degradation via thermal and oxidative mechanisms during processing [34].

An increase in drying temperature resulted in a pronounced reduction in TMA values. This trend was particularly evident in the HAD samples, where 70 °C HAD (1287.25 mg/kg dw) retained higher TMA levels compared with 80 °C HAD (908.36 mg/kg dw) and 60 °C HAD (775.82 mg/kg dw). These findings indicated that elevated temperatures accelerated the thermal degradation of anthocyanins, with losses becoming especially pronounced at 80 °C, consistent with findings that mild drying preserves TMA more effectively [29,35]. At identical drying temperatures, samples subjected to vacuum drying (150 mbar VD) exhibited higher TMA values compared with those dried under atmospheric conditions (HAD). Oxygen availability plays a key role in this process; vacuum drying limits oxidative degradation, whereas atmospheric drying conditions promote anthocyanin loss. For instance, TMA retention was greater in 70 °C VD (1313.89 mg/kg dw) than in 70 °C HAD (1287.25 mg/kg dw), and similarly in 80 °C VD (986.89 mg/kg dw) compared with 80 °C HAD (908.36 mg/kg dw). This observation may be attributed to the reduced oxygen availability under vacuum conditions, which limits oxidative degradation. Moreover, the lower effective boiling temperature under a vacuum likely decreases both the duration and intensity of heat exposure, thereby enhancing anthocyanin stability.

The protective effect of vacuum application was more pronounced at the intermediate temperature (70 °C), with the highest TMA value observed in the 70 °C, 150 mbar VD sample. In contrast, at lower temperatures (60 °C), prolonged drying times may have allowed enzymatic and oxidative reactions to persist, thereby contributing to increased TMA losses. Microwave treatments resulted in the lowest TMA values, particularly in the 360 W applied sample (463.13 mg/kg dw). This outcome suggested that high energy input induces localized temperature elevations, leading to rapid and extensive degradation of anthocyanins. In contrast, the 180 W MW treatment exhibited TMA losses comparable to those observed in the 60 °C HAD sample.

During digestion, TMA markedly decreased—undigested (2554.12 mg/kg) > gastric (1593.87 mg/kg) > intestinal (433.53 mg/kg) fractions—mirroring losses reported in aronia and other berries [50,76]. After gastric digestion, the TMA in fresh aronia fruit exhibited a decrease (37.60% relative to the undigested sample), whereas all dried samples showed an increasing trend (35.35–144.39%). The increase observed during the gastric phase in dried samples can be attributed to enhanced release of anthocyanins from the disrupted food matrix under acidic conditions, where the flavylium cation form is stabilized. This contrasting behavior may be attributed to matrix disruption during processing and acidic gastric conditions, which enhance the release and bioaccessibility of anthocyanins from the food matrix in dried samples. Furthermore, significant losses in TMA were observed after the intestinal phase, with reductions ranging from 30.53% to 82.93% relative to the undigested samples (*p* < 0.05) (Table 3), which might be related to the reduced stability and/or degradation of anthocyanins under intestinal conditions. In alignment with our observations, Tomas et al. [50] demonstrated a pronounced decrease in TMA content of aronia fruit, decreasing from 107.9 mg CGE/100 g fw in the undigested state to 9.4 mg CGE/100 g fw post-digestion. Notably, samples subjected to VD and MW treatments showed more pronounced increases in TMA during the gastric phase; nevertheless, significant losses were still observed during the intestinal phase. However, the intestinal phase leads to substantial degradation due to the instability of anthocyanins at neutral-to-alkaline pH, resulting in structural breakdown, oxidation, and conversion into phenolic acids and other degradation products. Similar reductions have been noted in plum, elderberry, and berry matrices after in vitro digestion [72]. The decline is due to the pH-dependent instability of anthocyanins: flavylium cations are stable in acidic gastric conditions but degrade under near-neutral intestinal pH, undergoing oxidation, structural breakdown, and interactions with enzymes and other food components [77].

### 3.6. Drying Kinetics of Aronia Fruits

#### 3.6.1. Effective Moisture Diffusivity (*D*_eff_)

*D*_eff_ was calculated and shown in Table 4. *D*_eff_ values increased with rising drying temperature and power levels across all methods. The lowest *D*_eff_ was observed in 60 °C HAD (1.05 × 10^−8^ m^2^/s), indicating the slowest moisture removal. In contrast, the highest diffusivity was obtained under 360 W MW (1.40 × 10^−7^ m^2^/s), confirming that microwave drying significantly enhanced internal mass transfer. Among all conditions, vacuum drying at 70 °C showed the highest model accuracy (R^2^ = 0.997), suggesting that vacuum-assisted drying not only improved moisture diffusion but also yielded consistent experimental data.

#### 3.6.2. Arrhenius Graphic

**Figure 3:**
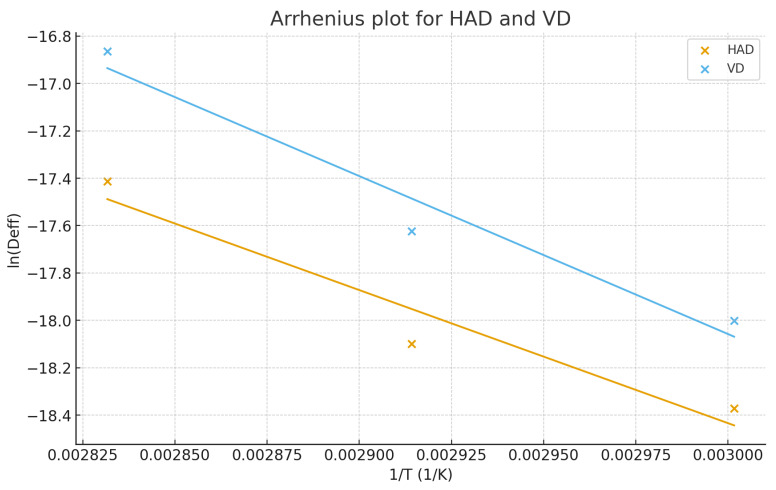
Arrhenius plot for hot air drying (HAD) and vacuum drying (VD).

An Arrhenius plot for hot air drying (HAD) and vacuum drying (VD) is depicted in Figure 3. The Arrhenius plot is commonly used to examine the temperature dependence of the effective moisture diffusivity (*D*_eff_). In this graph, ln(*D*_eff_) is plotted on the vertical axis, while 1/T (1/K) (1/Kelvin (K^−1^) is placed on the horizontal axis. As temperature increases, the 1/T values decrease, and in response, ln(*D*_eff_) increases, which confirms that moisture diffusion is accelerated at higher temperatures. The Arrhenius plot demonstrated that the effective moisture diffusivity (*D*_eff_) increased with rising temperature under both hot-air (HAD) and vacuum (VD) conditions, confirming that moisture transfer is a temperature-dependent phenomenon. This linear relationship between ln(*D*_eff_) and 1/T indicates that the drying process follows an Arrhenius-type behavior, as previously reported in several studies [78]. In agreement with our study, the effective moisture diffusivity (*D*_eff_) of pumpkin seeds increased with rising drying temperature, and the plot of ln(*D*_eff_) versus 1/T was linear, indicating Arrhenius-type behavior [79]. Notably, the increase in *D*_eff_ was more pronounced in VD, suggesting a greater sensitivity to temperature under reduced pressure conditions.

The superior performance of VD can be attributed to the lower atmospheric pressure, which decreases the resistance to mass transfer and facilitates the evaporation of moisture at lower temperatures. Reduced pressure conditions also promote structural disruption of cell walls, enhancing internal moisture mobility and accelerating diffusion [66]. As a result, higher *D*_eff_ values were obtained in vacuum drying compared to HAD at the same temperature level, which is consistent with prior findings that vacuum-assisted drying improves moisture transport efficiency and drying kinetics [80]. The results clearly indicated that the increase in ln(*D*_eff_) with temperature confirms the enhancement of the diffusion rate, both hot air (HA) and vacuum (V) drying methods exhibit Arrhenius-type behavior, and the increase is more pronounced in the vacuum drying method, suggesting a stronger influence of temperature on diffusion under reduced pressure conditions. Additionally, it was seen that vacuum drying was more effective. This results from the fact that vacuum conditions provide reduced pressure, which lowers the resistance to mass transfer. This also facilitates the disruption of cell walls, allowing internal moisture to migrate outward more rapidly. Therefore, at the same temperature level, *D*_eff_ values obtained in vacuum drying are higher compared to hot air drying, demonstrating that a vacuum significantly enhances moisture diffusion efficiency.

Consequently, as expected, the effective moisture diffusivity (*D*_eff_) increased with increasing temperature under both hot-air (HAD) and vacuum (VD) conditions. The Arrhenius plot exhibited an almost linear trend, indicating that the moisture diffusion during drying followed an Arrhenius-type behavior. This suggests that the drying process was temperature-dependent, and the activation energy for diffusion could be determined from the slope of the linear regression line.

#### 3.6.3. Activation Energy (Ea)

The temperature dependence of the effective moisture diffusivity (*D*_eff_) was analyzed using the Arrhenius equation, expressed as:

ln(*D*_eff_) = ln (*D*_0_) − Ea/Rx1/T

where *D*_0_ is the pre-exponential factor, Ea is the activation energy (kJ/mol), R is the universal gas constant (8.314 J/mol·K), and T is the absolute temperature (K). In this model, the slope of the ln(*D*_eff_) − 1/T regression line corresponds to −Ea/R, allowing the calculation of activation energy using the Arrhenius relationship, as commonly applied in drying studies [81].

Based on the linear regression slopes obtained from the Arrhenius plots (Figure 3), the activation energy (Ea) values were calculated as:

HAD: *Ea* ≈ 4.67 × 104 J/mol ≈ 46.7 kJ/mol Ea

VD: *Ea* ≈ 5.54 × 104 J/mol ≈ 55.4 kJ/mol Ea

The higher Ea value obtained for vacuum drying indicates that moisture diffusion under vacuum conditions is more temperature-dependent, which may be attributed to reduced ambient pressure and enhanced internal vapor transport [82,83]. Similar trends have been reported in previous studies, suggesting that vacuum-assisted drying promotes mass transfer efficiency by facilitating structural changes in the cellular matrix and reducing diffusion resistance [80,84]. The linearity of the Arrhenius plots further confirmed that both drying methods follow Arrhenius-type diffusion behavior, consistent with the literature findings [85].

The activation energy (Ea) values obtained from the Arrhenius model confirmed that moisture diffusion during drying was temperature-dependent for both the HAD and VD methods. The Ea value for vacuum drying (55.4 kJ/mol) was higher than that of hot air drying (46.7 kJ/mol), indicating that the moisture transfer mechanism in vacuum drying is more sensitive to temperature variations. This higher activation energy may be attributed to enhanced mass transfer under reduced pressure conditions, which facilitates moisture removal at lower temperatures [33,86].

Similar findings have been reported in previous studies, suggesting that vacuum-assisted drying might promote moisture diffusivity by lowering the boiling point of water and reducing mass transfer resistance [80,83]. Therefore, vacuum drying can be considered a more efficient drying method, particularly when rapid moisture removal and energy efficiency are desired.

### 3.7. Modeling of Drying Curves

Statistical results for dried fruits, including model coefficients, R^2^, RMSE, and χ^2^ values, are presented in Table 5. The R^2^, RMSE and χ2 values varied from 0.9043 to 1.000, 0.0017 to 0.0892, and 0.0000 to 0.1513 respectively. Considering the statistical criteria, the Midilli models—particularly under the 70 °C VD condition—were determined to be the most suitable drying models, exhibiting the highest coefficient of determination (R^2^ = 1.0000) and the lowest RMSE and χ^2^ values. This indicates an excellent agreement between the experimental and predicted moisture ratio values, confirming that the Midilli model provides the best fit for describing the drying behavior of the samples.

### 3.8. Principal Component Analysis (PCA)

**Figure 4:**
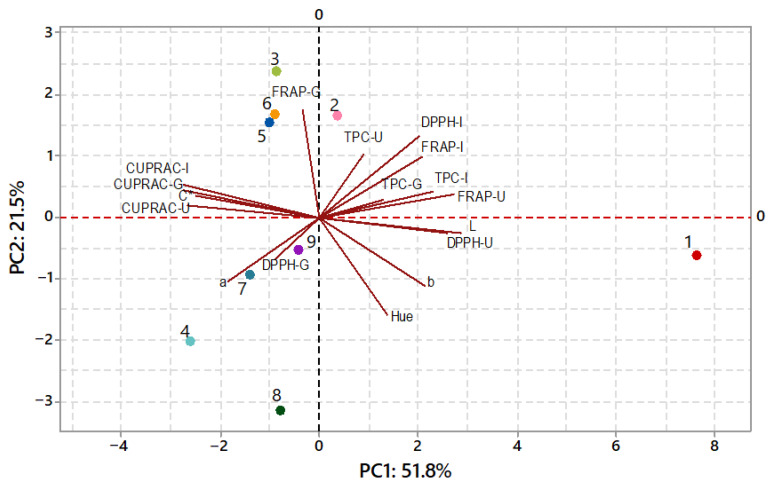
PCA biplot of fresh and dried aronia samples (1: fresh fruit; 2: 80 °C HAD (hot air drying); 3: 70 °C HAD; 4: 60 °C HAD; 5: 80 °C, 150 mbar VD (vacuum drying); 6: 70 °C, 150 mbar VD; 7: 60 °C, 150 mbar VD; 8: 360 W MW (microwave drying); 9: 180 W MW). TPC-U: total phenolic content (TPC) undigested; TPC-G: TPC gastric phase; TPC-I: TPC intestinal phase. The same labeling is used for DPPH, CUPRAC and FRAP. Color values: L, a, b, chroma (C*), and hue.

Principal component analysis was performed on the physicochemical and bioactive composition of the samples to better illustrate the variation among products subjected to different drying techniques. (Figure 4). Two principal components were sufficient to explain a substantial proportion of the total variability (PC1 = 51.8% and PC2 = 21.5%, explaining 73.3% of the total variance). In this context, the PCA biplot (Figure 4) elucidated the influence of processing conditions on both functional and color attributes of the samples. In the biplot, fresh aronia fruit was clearly distinguished along the positive PC1 axis, primarily due to its markedly higher undigested antioxidant capacities (DPPH-U and FRAP-U) and lightness (L), indicating a distinct biochemical profile compared with all dried products. Among the dried samples, conventional hot air drying (HAD) at 70 °C and 80 °C tended to cluster towards positive PC2 values, suggesting relatively higher retention of specific antioxidant activities compared with other drying techniques. Microwave drying (MW) at 360 W was located on the negative side of PC2, associated with lower post-digestion bioactive retention, while 180 W MW occupied an intermediate position. Vacuum drying treatments (VD) (60–80 °C under reduced pressure) were grouped near the center of the plot, reflecting intermediate physicochemical and bioactive profiles in terms of antioxidant measures and color parameters. Overall, these results indicated that drying technique and conditions significantly affected the compositional and functional characteristics of the processed samples, with conventional HAD favoring antioxidant preservation and microwave drying showing comparatively greater losses.

## 4. Conclusions

This study demonstrates that drying technology governs not only the retention of phenolic compounds but also their gastrointestinal behavior through process-induced structural modifications. The results indicate that phenolic functionality is primarily controlled by matrix transformation rather than absolute compound loss, emphasizing the importance of process–structure–function relationships in dried fruit systems. Among the tested conditions, vacuum drying at 70 °C and 150 mbar provided the most favorable balance between thermal exposure and oxygen limitation, resulting in improved phenolic stability and digestive resilience. These findings highlight vacuum-assisted drying as a targeted strategy for designing functional dried aronia products with enhanced bioactive performance.

Although the use of a standardized and well-established in vitro digestion model provides a controlled and reproducible framework for evaluating gastrointestinal behavior, it may not fully capture the complexity of in vivo physiological processes, including absorption and metabolism.

Future studies should validate these findings under physiological conditions and incorporate advanced microstructural analyses to better elucidate matrix–bioactive interactions. From an industrial perspective, the optimization and scale-up of vacuum-assisted drying may provide a promising route for the development of functional berry-based products with improved nutritional efficacy.

## Figures and Tables

**Table 1:** Color changes of dried aronia fruits.

Sample	L*	a*	b*	Chroma	Hue
Aronia fruit	21.36 ± 0.66 ^a^	0.06 ± 0.01 ^e^	−0.02 ± 0.01 ^a^	0.06 ± 0.02 ^f^	336.84 ± 7.37 ^a^
80 °C HAD	17.10 ± 0.01 ^b^	0.29 ± 0.05 ^cd^	−0.64 ± 0.03 ^c^	0.72 ± 0.04 ^d^	294.61 ± 2.69 ^cd^
70 °C HAD	13.39 ± 0.04 ^e^	0.27 ± 0.09 ^d^	−0.93 ± 0.07 ^de^	0.98 ± 0.09 ^bc^	286.91 ± 4.17 ^e^
60 °C HAD	11.46 ± 0.02 ^g^	0.74 ± 0.09 ^b^	−0.73 ± 0.07 ^c^	1.04 ±0.04 ^abc^	314.87 ± 4.36 ^b^
80 °C, 250 mbar VD	13.19 ± 0.01 ^e^	0.41 ± 0.09 ^c^	−1.02 ± 0.08 ^e^	1.10 ± 0.11 ^ab^	291.72 ± 2.71 ^cde^
70 °C, 150 mbar VD	11.60 ± 0.11 ^g^	0.30 ± 0.07 ^cd^	−0.87 ± 0.12 ^d^	0.92 ± 0.16 ^c^	288.67 ± 5.22 ^de^
60 °C, 150 mbar VD	12.18 ± 0.03 ^f^	0.88 ± 0.05 ^a^	−0.74 ± 0.11 ^c^	1.15 ± 0.03 ^a^	320.03 ± 4.55 ^b^
360 W MW	16.00 ± 0.02 ^d^	0.68 ± 0.03 ^b^	−0.33 ± 0.05 ^b^	0.75 ± 0.04 ^d^	334.37 ± 3.47 ^a^
180 W MW	16.54 ± 0.06 ^c^	0.18 ± 0.03 ^d^	−0.35 ± 0.04 ^b^	0.40 ± 0.05 ^e^	297.20 ± 5.64 ^c^

Different letters in the same column display significant differences (*p* < 0.05). Results are given as mean ± standard deviation (n = 3).

**Table 2:** TPC and TAC with DPPH, CUPRAC and FRAP assays of fresh and dried aronia fruits.

	Undigested	Gastric Phase	Intestinal Phase
TPC (mg GAE/100 g dw)			
Aronia fruit	640.41 ± 4.14 ^abcA^	192.00 ± 1.24 ^aC^	294.40 ± 1.90 ^aB^
80 °C HAD	644.73 ± 2.48 ^aA^	192.19 ± 6.53 ^aC^	285.74 ± 12.90 ^abB^
70 °C HAD	643.30 ± 12.29 ^abA^	188.14 ± 9.81 ^abC^	274.24 ± 1.50 ^bcB^
60 °C HAD	633.94 ± 3.39 ^abcA^	185.80 ± 1.68 ^abC^	261.44 ± 5.86 ^cB^
80 °C, 150 mbar VD	620.76 ± 3.33 ^deA^	181.29 ± 6.65 ^bC^	286.10 ± 14.00 ^abB^
70 °C, 150 mbar VD	630.30 ± 8.87 ^bcdA^	190.83 ± 3.78 ^aC^	275.62 ± 0.95 ^bB^
60 °C, 150 mbar VD	627.44 ± 0.70 ^cdA^	188.32 ± 5.79 ^abC^	274.83 ± 2.57 ^bcB^
360 W MW	606.85 ± 6.70 ^fA^	185.30 ± 3.93 ^abC^	280.60 ± 8.25 ^abB^
180 W MW	608.52 ± 14.20 ^efA^	193.59 ± 1.11 ^aC^	277.80 ± 11.31 ^bB^
TAC (µmol TE/g dw)			
DPPH			
Aronia fruit	168.97 ± 2.09 ^aA^	5.47 ± 0.61 ^aC*^	63.77 ± 5.78 ^aB^
80 °C HAD	13.19 ± 0.35 ^bcB^	5.39 ± 0.25 ^aC^	55.43 ± 1.56 ^bA^
70 °C HAD	11.76 ± 0.76 ^dB^	5.65 ± 0.12 ^aC^	56.01 ± 4.12 ^bA^
60 °C HAD	13.41 ± 0.18 ^bB^	5.62 ± 0.11 ^abC^	37.19 ± 1.61 ^dA^
80 °C, 150 mbar VD	11.97 ± 0.09 ^cdB^	5.38 ± 0.17 ^bC^	55.08 ± 2.08 ^bA^
70 °C, 150 mbar VD	11.51 ± 0.40 ^dB^	5.85 ± 0.23 ^aB^	55.77 ± 5.50 ^bA^
60 °C, 150 mbar VD	12.03 ± 0.09 ^cdB^	5.54 ± 0.15 ^abC^	48.79 ± 2.09 ^cA^
360 W MW	14.24 ± 0.13 ^bB^	5.89 ± 0.13 ^aC^	40.70 ± 2.92 ^dA^
180 W MW	13.30 ± 0.02 ^bB^	5.80 ± 0.04 ^abC^	51.65 ± 1.89 ^bcA^
CUPRAC			
Aronia fruit	825.24 ± 92.22 ^bA^	53.04 ± 3.62 ^dB^	87.53 ± 8.00 ^cA^
80 °C HAD	1366.32 ± 176.67 ^aA^	377.70 ± 8.92 ^bcB^	552.20 ± 67.29 ^abB^
70 °C HAD	1365.27 ± 176.96 ^aA^	409.28 ± 15.32 ^abC^	624.72 ± 27.96 ^aB^
60 °C HAD	1451.84 ± 354.18 ^aA^	383.29 ± 29.29 ^bcB^	555.18 ± 73.05 ^abB^
80 °C, 150 mbar VD	1330.63 ± 68.29 ^aA^	407.96 ± 8.47 ^abC^	526.36 ± 20.09 ^abB^
70 °C, 150 mbar VD	1252.85 ± 108.90 ^aA^	394.10 ± 41.98 ^abcB^	585.87 ± 136.64 ^abB^
60 °C, 150 mbar VD	1309.23 ± 203.80 ^aA^	358.56 ± 27.94 ^bcB^	518.58 ± 89.42 ^abB^
360 W MW	1246.91 ± 159.86 ^aA^	349.79 ± 12.67 ^cB^	495.36 ± 51.29 ^bB^
180 W MW	1478.14 ± 195.43 ^aA^	440.97 ± 63.63 ^aB^	573.77 ± 37.92 ^abB^
FRAP			
Aronia fruit	419.32 ± 12.09 ^aA^	58.18 ± 1.55 ^fC^	106.03 ± 6.75 ^aB^
80 °C HAD	166.41 ± 17.22 ^cA^	115.89 ± 0.93 ^bB^	37.39 ± 9.81 ^dC^
70 °C HAD	225.87 ± 22.74 ^bA^	126.38 ± 3.92 ^aB^	67.09 ± 27.45 ^cC^
60 °C HAD	116.78 ± 19.20 ^eA^	30.74 ± 2.30 ^gB^	19.17 ± 8.08 ^fC^
80 °C, 150 mbar VD	147.68 ± 14.21 ^cdA^	102.44 ± 3.69 ^cB^	71.02 ± 13.28 ^bC^
70 °C, 150 mbar VD	143.93 ± 14.73 ^cdeA^	118.81 ± 7.14 ^bB^	75.71 ± 11.77 ^bC^
60 °C, 150 mbar VD	148.56 ± 24.35 ^cdA^	71.17 ± 1.86 ^eB^	35.40 ± 8.36 ^dC^
360 W MW	88.93 ± 8.34 ^fA^	55.70 ± 3.79 ^fB^	22.03 ± 0.54 ^fC^
180 W MW	131.29 ± 9.34 ^dA^	77.79 ± 1.95 ^dB^	27.80 ± 0.10 ^eC^

Different lower case letters in the column and capital letters in the row are significantly different (*p* < 0.05). Results are given as mean ± standard deviation (n = 3). * indicates non-significance (*p* > 0.05) (in DPPH gastric phase).

**Table 3:** Total monomeric anthocyanin (TMA) content of fresh and dried aronia fruits.

TMA (mg C3G/kg dw *)	Undigested	Gastric Phase	Intestinal Phase
Aronia fruit	2554.12 ± 30.53 ^aA^	1593.87 ± 2.01 ^dB^	435.53 ± 5.41 ^cC^
80 °C HAD	908.36 ± 11.05 ^dB^	1694.14 ± 14.47 ^cdA^	333.51 ± 0.49 ^dC^
70 °C HAD	1287.25 ± 2.85 ^bcB^	1742.30 ± 13.06 ^cdA^	449.75 ± 4.57 ^cC^
60 °C HAD	775.82 ± 8.72 ^deB^	1413.35 ± 7.24 ^eA^	211.15 ± 3.31 ^eC^
80 °C, 150 mbar VD	986.89 ± 20.71 ^cdB^	2158.57 ± 7.16 ^bA^	553.51 ± 3.60 ^bC^
70 °C, 150 mbar VD	1313.89 ± 3.29 ^bB^	2501.71 ± 7.09 ^aA^	694.62 ± 1.41 ^aC^
60 °C, 150 mbar VD	1003.86 ± 29.57 ^bcdB^	1856.06 ± 12.75 ^cA^	573.59 ± 6.32 ^bC^
360 W MW	463.13 ± 15.50 ^eB^	1132.03 ± 12.35 ^fA^	321.69 ± 1.12 ^dB^
180 W MW	771.71 ± 19.50 ^deB^	1760.31 ± 12.95 ^cdA^	366.77 ± 1.62 ^dC^

Different lowercase letters in the same column indicate significant differences between treatments, while uppercase letters indicate alterations that occur before and during the gastric and intestinal digestion steps (*p* < 0.05). Results are given as mean ± standard deviation (n = 3). * C3G: cyanidin-3-glucoside equivalents.

**Table 4:** *D*_eff_ of the dried aronia fruits.

Drying Parameters	*D*_eff_ (m^2^/s)	R^2^
60 °C HAD	1.05 × 10^−8^	0.975
70 °C HAD	1.38 × 10^−8^	0.983
80 °C HAD	2.74 × 10^−8^	0.977
60 °C, 150 mbar VD	1.52 × 10^−8^	0.995
70 °C, 150 mbar VD	2.22 × 10^−8^	0.997
80 °C, 150 mbar VD	4.74 × 10^−8^	0.993
180 W MW	2.64 × 10^−8^	0.979
360 W MW	1.40 × 10^−7^	0.991

**Table 5:** Statistical results obtained from the modeling of aronia fruits.

Model			60 °C HAD	70 °C HAD	80 °C HAD	60 °C, 150 mbar VD	70 °C, 150 mbar VD	80 °C, 150 mbar VD	180 W MW	360 W MW
Page	Model coefficient	n	1.0819	1.0303	1.2330	1.0517	0.9648	1.1087	0.7896	0.9733
		k	0.0016	0.0033	0.0020	0.0033	0.0076	0.0079	0.0251	0.0451
	R^2^		0.9871	0.9833	0.9915	0.9906	0.9959	0.9929	0.9819	0.9987
	RMSE		0.0340	0.0396	0.0302	0.0312	0.0215	0.0268	0.0389	0.0085
	χ^2^		0.0139	0.0298	0.0100	0.0097	0.0037	0.0072	0.0287	0.0024
Modified Page	Model coefficient	n	1.0819	1.0303	1.2330	1.0517	0.9648	1.1087	0.7896	0.9733
		k	0.0027	0.0039	0.0066	0.0044	0.0064	0.0127	0.0094	0.0414
	R^2^		0.9871	0.9833	0.9915	0.9906	0.9959	0.9929	0.9819	0.9987
	RMSE		0.0340	0.0396	0.0302	0.0312	0.0215	0.0268	0.0389	0.0085
	χ^2^		0.0139	0.0298	0.0100	0.0097	0.0037	0.0072	0.0287	0.0024
Logarithmic	Model coefficient	k	0.0017	0.0023	0.0044	0.0033	0.0058	0.0099	0.0087	0.0407
		a	1.2387	1.2109	1.2140	1.0935	1.0034	1.1013	0.9015	0.9855
		c	−0.2781	−0.2699	−0.2271	−0.1240	−0.0250	−0.1186	0.0192	0.0014
	R^2^		0.9959	0.9947	0.9987	0.9961	0.9966	0.9980	0.9723	0.9987
	RMSE		0.0201	0.0228	0.0125	0.0211	0.0211	0.0152	0.0493	0.0088
	χ^2^		0.0044	0.0094	0.0016	0.0040	0.0031	0.0021	0.0438	0.0025
Lewis	Model coefficient	k	0.0027	0.0039	0.0070	0.0044	0.0063	0.0132	0.0092	0.0409
	R^2^		0.9851	0.9830	0.9803	0.9899	0.9956	0.9903	0.9582	0.9985
	RMSE		0.0351	0.0390	0.0440	0.0309	0.0210	0.0300	0.0575	0.0092
	χ^2^		0.0160	0.0304	0.0232	0.0105	0.0040	0.0099	0.0661	0.0029
Henderson and Pabis	Model coefficient	k	0.0027	0.0038	0.0072	0.0044	0.0062	0.0133	0.0082	0.0405
		a	1.0000	0.9849	1.0333	0.9984	0.9860	1.0120	0.9134	0.9858
	R^2^		0.9851	0.9834	0.9817	0.9899	0.9959	0.9905	0.9721	0.9987
	RMSE		0.0365	0.0395	0.0443	0.0324	0.0216	0.0312	0.0482	0.0086
	χ^2^		0.0160	0.0296	0.0216	0.0105	0.0037	0.0097	0.0442	0.0025
Wang–Singh	Model coefficient	b	−0.0021	−0.0030	−0.0049	−0.0033	−0.0045	−0.0094	−0.0069	−0.0288
		a	1.15 × 10^−6^	2.35 × 10^−6^	6.12 × 10^−6^	2.89 × 10^−6^	5.31 × 10^−6^	2.30 × 10^−5^	1.28 × 10^−5^	0.0002
	R^2^		0.9893	0.9810	0.9968	0.9862	0.9760	0.9905	0.9043	0.9736
	RMSE		0.0309	0.0422	0.0184	0.0378	0.0522	0.0311	0.0892	0.0388
	χ^2^		0.0115	0.0338	0.0037	0.0143	0.0218	0.0097	0.1513	0.0498
Weibullian I	Model coefficient	β	1.0819	1.0303	1.2330	1.0517	0.9648	1.1087	0.7896	0.9733
		α	375.2553	257.9996	152.5004	228.9016	156.9039	78.5059	106.3766	24.1378
	R^2^		0.9871	0.9833	0.9915	0.9906	0.9959	0.9929	0.9819	0.9987
	RMSE		0.0340	0.0396	0.0302	0.0312	0.0215	0.0268	0.0389	0.0085
	χ^2^		0.0139	0.0298	0.0100	0.0097	0.0037	0.0072	0.0287	0.0024
Weibullian II	Model coefficient	n	1.0819	1.0303	1.2330	1.0517	0.9648	1.1087	0.7896	0.9733
		δ	811.1730	579.6526	299.9499	505.8765	372.4544	166.5744	305.9158	56.8665
	R^2^		0.9871	0.9833	0.9915	0.9906	0.9959	0.9929	0.9819	0.9987
	RMSE		0.0340	0.0396	0.0302	0.0312	0.0215	0.0268	0.0389	0.0085
	χ^2^		0.0139	0.0298	0.0100	0.0097	0.0037	0.0072	0.0287	0.0024
Midilli I	Model coefficient	k	0.0100	0.0169	0.0073	0.0139	0.0157	0.0242	0.0513	0.0474
		a	0.9976	1.0037	0.9961	0.9996	0.9988	0.9998	1.0216	0.9965
		b	−0.0004	−0.0006	−0.0005	−0.0004	−0.0002	−0.0009	−0.0005	−0.0001
		n	0.6823	0.6364	0.9251	0.7267	0.7999	0.7952	0.5971	0.9521
	R^2^		0.9989	0.9993	0.9989	0.9998	0.9996	1.0000	0.9904	0.9989
	RMSE		0.0110	0.0085	0.0122	0.0053	0.0079	0.0022	0.0298	0.0083
	χ^2^		0.0012	0.0012	0.0013	0.0002	0.0004	0.0000	0.0151	0.0021
Modified Midilli I	Model coefficient	k	0.0104	0.0160	0.0076	0.0140	0.0158	0.0243	0.0447	0.0484
		b	−0.0004	−0.0006	−0.0005	−0.0004	−0.0002	−0.0009	−0.0005	−0.0001
		n	0.6754	0.6458	0.9162	0.7260	0.7984	0.7949	0.6218	0.9468
	R^2^		0.9989	0.9993	0.9988	0.9998	0.9996	1.0000	0.9901	0.9989
	RMSE		0.0105	0.0083	0.0117	0.0050	0.0073	0.0020	0.0295	0.0082
	χ^2^		0.0012	0.0012	0.0014	0.0002	0.0004	0.0000	0.0157	0.0022
Modified Midilli II	Model coefficient	k	0.0025	0.0028	0.0062	0.0087	0.0150	0.0187	0.0346	0.0474
		a	4.1999	6.2370	1.3708	1.8282	1.1716	1.4686	1.5928	1.0087
		b	−3.2025	−5.2338	−0.3742	−0.8284	−0.1725	−0.4687	−0.5723	−0.0119
		n	0.6971	0.6436	0.9013	0.7056	0.7738	0.7682	0.5734	0.9478
	R^2^		0.9989	0.9993	0.9990	0.9998	0.9997	1.0000	0.9898	0.9989
	RMSE		0.0107	0.0086	0.0115	0.0052	0.0070	0.0017	0.0308	0.0083
	χ^2^		0.0011	0.0012	0.0012	0.0002	0.0003	0.0000	0.0161	0.0021
Aghbashlo	Model coefficient	k1	0.0023	0.0034	0.0050	0.0039	0.0063	0.0109	0.0118	0.0420
		k2	−0.0004	−0.0004	−0.0012	−0.0004	−2.16 × 10^−5^	−0.0014	0.0020	0.0006
	R^2^		0.9913	0.9865	0.9969	0.9929	0.9956	0.9955	0.9732	0.9986
	RMSE		0.0279	0.0356	0.0181	0.0272	0.0223	0.0214	0.0472	0.0089
	χ^2^		0.0093	0.0240	0.0036	0.0074	0.0040	0.0046	0.0424	0.0026

## Data Availability

The original contributions presented in this study are included in the article. Further inquiries can be directed to the corresponding author.
